# Transcriptional analysis of the *HeT-A *retrotransposon in mutant and wild type stocks reveals high sequence variability at Drosophila telomeres and other unusual features

**DOI:** 10.1186/1471-2164-12-573

**Published:** 2011-11-23

**Authors:** David Piñeyro, Elisenda López-Panadès, María Lucena-Pérez, Elena Casacuberta

**Affiliations:** 1 Institute of Evolutionary Biology (CSIC-UPF), Passeig Marítim de la Barceloneta 37-49, 08003, Barcelona, Spain

## Abstract

**Background:**

Telomere replication in Drosophila depends on the transposition of a domesticated retroelement, the *HeT-A *retrotransposon. The sequence of the *HeT-A *retrotransposon changes rapidly resulting in differentiated subfamilies. This pattern of sequence change contrasts with the essential function with which the *HeT-A *is entrusted and brings about questions concerning the extent of sequence variability, the telomere contribution of different subfamilies, and whether wild type and mutant Drosophila stocks show different *HeT-A *scenarios.

**Results:**

A detailed study on the variability of *HeT-A *reveals that both the level of variability and the number of subfamilies are higher than previously reported. Comparisons between GIII, a strain with longer telomeres, and its parental strain Oregon-R indicate that both strains have the same set of *HeT-A *subfamilies. Finally, the presence of a highly conserved splicing pattern only in its antisense transcripts indicates a putative regulatory, functional or structural role for the *HeT-A *RNA. Interestingly, our results also suggest that most *HeT-A *copies are actively expressed regardless of which telomere and where in the telomere they are located.

**Conclusions:**

Our study demonstrates how the *HeT-A *sequence changes much faster than previously reported resulting in at least nine different subfamilies most of which could actively contribute to telomere extension in Drosophila. Interestingly, the only significant difference observed between Oregon-R and GIII resides in the nature and proportion of the antisense transcripts, suggesting a possible mechanism that would in part explain the longer telomeres of the GIII stock.

## Background

Drosophila has a unique mechanism of telomere maintenance. Instead of using the telomerase holoenzyme as most eukaryotes, Drosophila replenishes the telomeres by specific transpositions onto the end of the chromosomes of three retrotransposons, *HeT-A*, *TART *and *TAHRE *[1,2]. The telomeric retrotransposons are completely excluded from euchromatin and share unique characteristics, possibly linked to their telomeric role, that separate them from their non-LTR counterparts. Orthologues of *HeT-A *and *TART *have been cloned and studied from species more than 60 MY distant (*D.melanogaster *- *D.virilis*), demonstrating that the telomeric retrotransposons predate the separation of the extant species as well as the robustness and reliability of this mechanism of telomere maintenance [3,4]. Surprisingly, *HeT-A *and *TART *orthologues, although committed to the essential function of telomere replication, are far from being static, and while maintaining their basic structures allow their sequence to change rapidly, evolving faster than euchromatic genes and other retrotransposons [5]. This trend of fast sequence change also results in differences within the same Drosophila species and for the *D. melanogaster HeT-A *element two previous studies have suggested the presence of a small number of subfamilies coexisting in the same stock [6,7].

Previous studies have attempted to classify the genomic copies of the *HeT-A *element in several subfamilies according to their variability in the 3'UTR [6] and also in the ORF [7]. These studies found four subfamilies considering ORF variability and two considering 3'UTR variability. Taking into account that those studies were based in a limited number of genomic copies, our first objective was to perform an exhaustive survey at genomic level in order to obtain a more accurate picture of the real variability of the *HeT-A *element.

Other retroelements also form subfamilies in a given genome, as for example Tnt1 in tobacco and L1 in mammalian genomes [8,9]. In the case of Tnt1, the different subfamilies have acquired different sequences at their regulatory regions that ensure the expression of a particular subfamily in response to different external factors, widening and diversifying in that way the number of opportunities for transposition [10]. In the case of L1, although remnants of several subfamilies exist in a given genome, only one subfamily seems to be active at a time [11]. Whether the existence of different *HeT-A *subfamilies has a putative role related to its own survival as a retrotransposon or to its telomeric function is still unknown. Studies comparing the number and dynamics of the different subfamilies between wild type and telomeric mutant stocks are needed to answer this question.

With the completion of the heterochromatic genome project [12] and the assembly of some telomeres for the particular Drosophila strain used in the sequencing project (isogenic strain 2057 yellow (*y^1^*); cinnabar (*cn^1^*) brown (*bw^1^*) speck (*sp^1^*) [7,13]) it was possible to obtain the first detailed view of the telomere structure in *Drosophila melanogaster*. Because the telomeric retrotransposons suffer from terminal erosion while being at the end of the chromosome, 5' truncated copies were expected. These two studies actually revealed that more *HeT-A *copies in the telomeric arrays have maintained ORFs and other regions needed for function than had originally been expected. The existence of functional copies in proximal regions of these long telomere arrays suggests that these interior sequences may be renewed more frequently than previously thought. In this case, the turnover in these arrays does not simply replace terminal sequence lost in DNA replication but is also necessary for rebuilding a large fraction of the telomere when needed [14]. If this were the case, it would be even more important to keep a fair number of *HeT-A *copies capable of active transposition to replenish or make up new telomeres whenever needed. Alternatively, full length elements in the middle of the array could also be explained by the simultaneous transposition of more than one *HeT-A *element, or access of *HeT-A *transposition intermediates to the end of the chromosome when terminal erosion has not yet taken place, for example when the capping complex is disassembled. Finding which *HeT-A *copies are actively being transcribed and whether transcriptional differences exist in response to subfamily affiliation or specific position in the telomeric array would help to better understand telomere biology in Drosophila.

Besides specialized structures composed of repeated sequences, telomeres are also composed of specific proteins and RNA [15]. Because Drosophila telomere elongation is dependent on elements located within the telomere itself, the presence of RNA at telomeres is not a surprise. Some years ago a non-coding antisense RNA containing telomeric repeats was reported in different mammals and named TERRA (Telomeric-Repeat containing RNA) [16]. TERRA RNA seems to be an important component of the telomeric heterochromatin with different regulatory functions in chromatin as well as direct regulation of the telomerase activity. Similarly to TERRA RNA, the telomeric retrotransposons *HeT-A *and *TART *are also transcribed from the antisense strand [17,18] and a recent report strongly suggests that short RNAs coming from the telomeres might have a role in telomere function, protection and development in Drosophila [19]. Therefore, with the new discoveries of non-coding RNAs being involved in different regulatory functions, as mentioned above, further studies on the potential role for the antisense RNAs of *HeT-A *and *TART *at Drosophila telomeres become crucial.

The work presented here aims at better characterizing the extent of *HeT-A *variability and determining if different *HeT-A *subfamilies could become more successful in a given stock. For this, we have chosen two different stocks, a wild type stock, Oregon-R, and a mutant stock with longer telomeres, the GIII stock bearing an Oregon-R background where the third chromosome from the *Tel-1 *mutant was introduced almost 10 years ago [20]. Although the cytological position of the *Tel-1 *mutation has been determined (3L (69)), it is still unknown the molecular cause responsible for the extremely long telomeres of the GIII stock (approximately ten times longer than in any wild type stock). We have amplified and sequenced from DNA and RNA sources in these two stocks some of the most variable regions inside the *HeT-A *sequence, and have been able to identify all previously defined *HeT-A *subfamilies. Moreover we describe five previously unreported *HeT-A *families, demonstrating that *HeT-A *variability is even greater than expected. Our results show that most *HeT-A *subfamilies are actively transcribed and some of the found variations allow us to draw the recent history for a number of *HeT-A *copies. Finally, we have done a wide study on *HeT-A *antisense transcription and found that most antisense transcripts suffer different alternative splicing with remarkable conservation. Interestingly, we find that the GIII and Oregon-R stocks mainly differ in which subfamilies contribute the most to antisense transcription. This leads us to suggest that due to the importance of non-coding RNAs in gene and heterochromatin regulation, the reported differences could explain in part the greater expression of *HeT-A *and ultimately the longer telomeres of the GIII stock.

## Results

### *HeT-A *sequence variability

**Figure 1:**
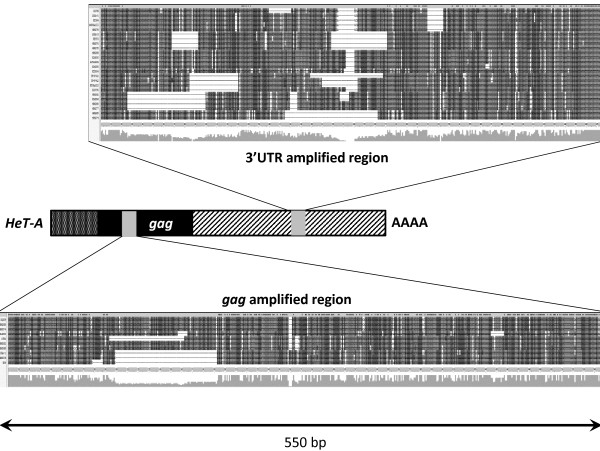
***HeT-A *regions amplified in this study**. Middle, schematic drawing of the *HeT-A *complete element. Upper and lower, nucleotide alignment of the amplified fragments from the *gag *gene and the 3'UTR using all previously sequences of the *HeT-A *elements from *Drosophila melanogaster *containing this region available in the data base. Sequences corresponding to the fragment inside the *gag *gene (from upper to lower): 01D09, 4R6265, 4R6274, XL4800, 17B3, 4R6262, 4R6268, 23Zn-1, 4R6276 and 9D4. Sequences from the fragment inside the 3'UTR region (from upper to lower): 1187X, 3Zn-1, RT394, 44PChrIII, 4R6276, 23Zn-3, RT473, 4R6270, 4R6278, 4R6269, XL4800, AJ549609, XL6255, XL5504, CS-T-A1, CS-T-A2, HTChrIII, XL4795, 4R6262, XL6256, 4R6268, 4R6277, 4R6265 and 4R6274. For correspondence of sequence name with the accession numbers see Additional file 2.

**Figure 2:**
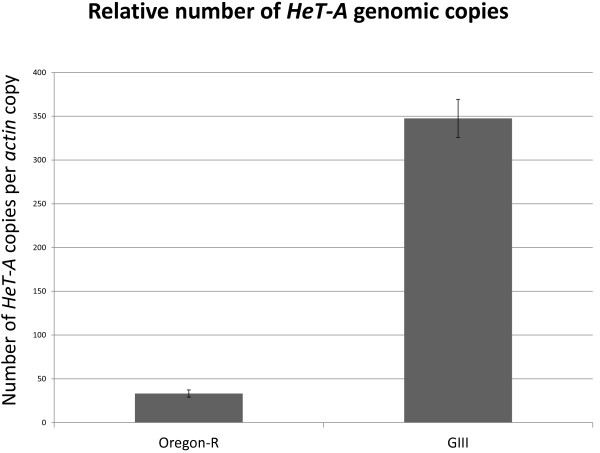
***HeT-A *genomic copy number**. Relative number of *HeT-A *genomic copies in the analyzed Drosophila stocks, Oregon-R and GIII. *HeT-A *copy number obtained by qPCR (using primers annealing at the 3'UTR) and normalized per copy number of the *actin *gene.

**Figure 3:**
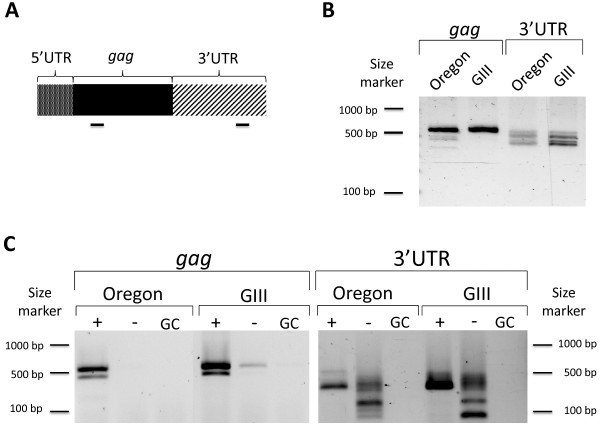
**Genomic and RNA amplification**. **A**. Schematic representation of a *HeT-A *element containing the 5'UTR (dotted box), the *gag *gene (black box) and the 3'UTR (lined box). Black bars at the bottom mark the amplified fragments. **B**. Genomic amplification of the indicated fragments in both, Oregon-R and GIII. Note the size variability of the amplified products indicative of size polymorphism among different *HeT-A *genomic copies. **C**. RNA amplification of the indicated fragment in both, Oregon-R and GIII stocks. +/- indicate specific amplification of sense and antisense strand respectively. GC indicates control for genomic DNA amplification.

**Figure 4:**
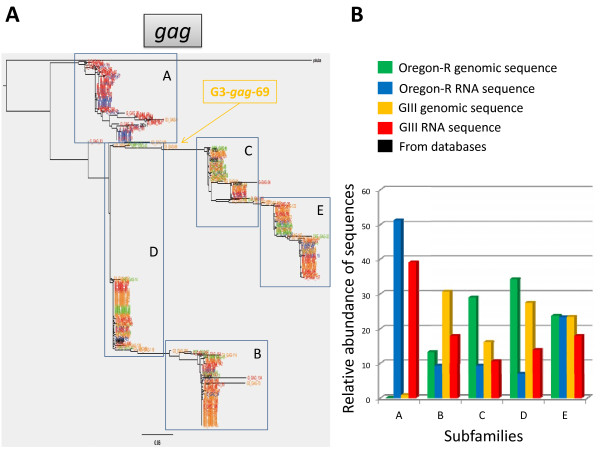
**Phylogenetic analysis of the sequences amplified for the *gag *fragment**. All amplified sequences from genomic DNA of Oregon-R and GIII (names in green and orange, respectively) and from total RNA from 3^rd ^instar larvae of Oregon-R and GIII (names in blue and red, respectively) were used. Also included are the previously annotated *HeT-A *sequences containing the analyzed fragment (shown in black). **A**. Phylogenetic tree constructed using all obtained sequences. The different subfamilies are indicated with boxes with the corresponding letter. Additionally, the tree includes a *Drosophila yakuba HeT-A *sequence (AF043258, named *yakuba *in the tree), in order to illustrate the degree of variability of *HeT-A *inside species and between species (*D.yakuba *and *D.melanogaster *are considered to be ~10 million years of genetic distance; source: http://flybase.org). The position of sequence G3-*gag*-69 is indicated. **B**. Bar chart representing the relative abundance of each subfamily.

**Figure 5:**
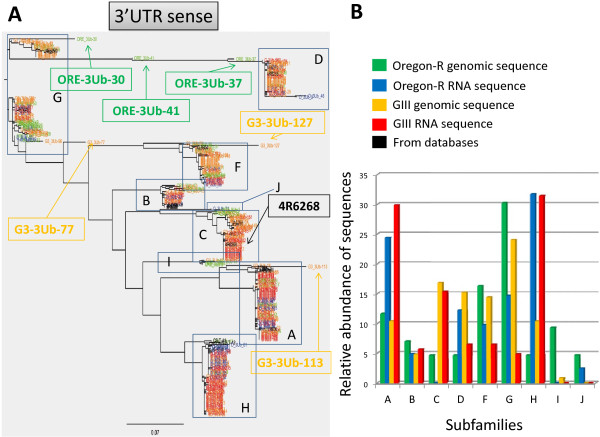
**Phylogenetic analysis of the sense sequences amplified for the 3'UTR fragment**. All amplified sequences from genomic DNA of Oregon-R and GIII (names in green and orange, respectively) and from total RNA from 3^rd ^instar larvae of Oregon-R and GIII (names in blue and red, respectively) were used. Also included are the previously annotated *HeT-A *sequences containing the analyzed fragment (shown in black). **A**. Phylogenetic tree constructed using all obtained sequences. The different subfamilies are indicated with boxes and corresponding letter. The position of sequences ORE-3Ub-30, ORE-3UB-41, ORE-3Ub-37, G3-3Ub-77, G3-3Ub-127, G3-3Ub-113 and the previously known 4R6268 are indicated. **B**. Bar chart representing the relative abundance of each subfamily.

Available information on telomere composition in Drosophila is partial (Additional files 1, 2); in order to investigate the nature and the extent of sequence variability in *HeT-A*, we decided to carry out a genome wide study analyzing two different regions along the retrotransposon, inside the *HeT-A *ORF (*gag*) and the 3' Untranslated Region (UTR). For this, we designed primers that would anneal to highly conserved sequences among copies, maximizing the number of different subfamilies that would be amplified, and in turn flanking highly variable regions in order to maximize the obtained information. Highly variable regions were selected by performing alignments of the amplified fragment from all *HeT-A *sequences already present in the databases in which the primers were contained (Figure 1 and Additional file 2). We first used the 3'UTR primers to obtain by qPCR an approximate copy number of *HeT-A *(Figure 2). We did obtain a ten-fold difference between the Oregon-R and the GIII strain. We then proceed to amplify both the *gag *and the 3'UTR fragment from genomic DNA. As expected from the alignment presented in Figure 1, the DNA fragments amplified for the 3'UTR region show a considerable size variation reflected in several amplified bands while amplified fragments for the ORF region display mainly two discrete bands in the case of Oregon DNA (Figure 3B). We cloned and sequenced the amplified fragments for the two regions, performed alignments and the resulting trees are shown in Figures 4A and 5A (copies from genomic DNA shown in green). The total number of analyzed sequences for the *gag *and the 3'UTR fragment is shown in Table 1.

**Table 1:** Number of sequences obtained and analyzed in our study.

	Genomic gag	Genomic 3'UTR	RNA gag sense	RNA 3'UTR sense	RNA 3'UTR antisense
**Oregon-R**	44	43	44	41	40

**GIII**	125	125	124	124	124

Taking into account the genomic copy number obtained for each strain (see Figure 1), the coverage is slightly higher than 1 for Oregon-R and slightly lower than 0.5 for GIII. Antisense strand amplification was performed only in the case of the 3'UTR due to its relative abundance.

As a result of amplifying the *gag *gene and the 3'UTR separately we were not able to know whether they belonged to the same copy; we thus used the complete *HeT-A *copies available in the database, and included them in our analysis. As several complete *HeT-A *copies present in the database belong to different subfamilies, we can in this manner observe if copies belonging to the same subfamily group together for both, the *gag *and the 3'UTR fragment. We chose a threshold of 10% difference to define the different subfamilies, as this was the minimum divergence percentage that allowed correlating our results with previous reports [6,7]. In the present study, we have found representatives of all the previously described subfamilies, for both the *gag *and the 3'UTR fragments (Figures 4A and 5A). Families A, B, C and D for the *gag *gene and families A and G for the 3'UTR region had been described previously [7,6]. Furthermore, our analysis has revealed greater variability in the *HeT-A *sequence identifying one new subfamily for the *gag *fragment and seven new subfamilies for the 3'UTR fragment, with a total of five (**A **to **E**) and nine (**A **to **J **minus **E**) subfamilies attending to *gag *and 3'UTR respectively. In spite of being full length, some of the previously classified *HeT-A *sequences, had only been analyzed for the *gag *or the 3'UTR region. Interestingly, our study has now found that the phylogenetic relationship among subfamilies is maintained for both fragments and therefore families **A **to **D **for the *gag *fragment correspond to families **A **to **D **for the 3'UTR fragment (Figures 4 and 5). With this result, our study integrates all previously described *HeT-A *subfamilies.

Unfortunately, this comparison is limited by the low amount of preexisting sequences containing both the *gag *and the 3'UTR fragment. This is the case of subfamily **E **present in the *gag *analysis, which does not have a corresponding subfamily **E **for the 3'UTR because no previous full-length *HeT-A *sequence belonging to this subfamily exists in the database. Finally, we should mention that the 10% of divergence does not take into account the gaps present inside sequences; thereby the high variability reported here is still a sub-estimation of the real variability inside the *HeT-A *sequences.

### *HeT-A *content and variability in the GIII strain versus Oregon-R

*HeT-A *content determined by qPCR (Figure 2) is approximately ten times higher in the GIII strain than in the wild type strain, Oregon-R. We wondered if this ten-fold difference in number of copies would result in higher variability. In order to investigate this question we analyzed three times more sequences of the *gag *and the 3'UTR fragments from the GIII strain (Table 1) and added them to the phylogenetic trees corresponding to each fragment (Figure 4A and 5A). As Figures 4A and 5A show, all genomic sequences from GIII (yellow) group together with the preexisting subfamilies from Oregon-R (green). There are a few cases in both fragments where sequences from both Oregon-R and GIII appear isolated between subfamilies. This is the case, for example, for the GIII sequence ***G3-Gag-89 ***in the *gag *tree (in yellow, Figure 4A), or sequences in the 3'UTR tree (Figure 5A), ***ORE-3Ub-30, ORE-3Ub-41 ***and ***ORE-3Ub-37 ***which are intermediates between the **G **and the **D **subfamilies, ***G3-3Ub-77 ***that has diverged from the **G **subfamily, or ***G3-3Ub-127 ***and ***G3-3Ub-113 ***which have respectively diverged from the **F **and **A **subfamilies.

Figures 4B and 5B reflect more graphically the relative abundance of each subfamily in the pool of sequences analyzed for each strain. With one exception, subfamily **J**, the content of each subfamily in Oregon-R and GIII is similar or proportional (attending to the fact that there are ten times more copies of *HeT-A *in GIII). The absence of subfamily **J **in GIII might be due to the insufficient coverage of our approach, or a lower fitness of this subfamily as suggested by its low presence also in Oregon-R. Alternatively, the absence of the **J **family in GIII suggests that the major increase in *HeT-A *copy number in this stock may be the result of an increase of only the telomeric copies of the parental stock Oregon-R. Previous studies have reported few *HeT-A *defective copies outside the telomeres in the heterochromatic region of the III and Y centromeres [21-23]. These small *HeT-A *fragments mostly correspond to the 3'UTR and have not been previously classified in subfamilies. All the sequences, from subfamilies **I **and **J **collected in this study show 99% or higher sequence identity with the centromeric sequences previously reported, suggesting that sequences belonging to these subfamilies come from non-telomeric regions.

Taking into account the total number of *HeT-A *copies obtained in the Oregon-R and GIII stocks (Figure 2) and the number of analyzed sequences (Table 1), the coverage presented here is slightly higher than 1 for the Oregon-R and slightly lower than 0.5 for the GIII stock. Although this coverage is low for a detailed analysis of each *HeT-A *copy present in these stocks, it allows a fair glance to the *HeT-A *content and variability in these two stocks. The results from Figures 4 and 5 indicate that in major terms, the higher number of *HeT-A *copies in GIII is consequence of the general and proportional increase of the different *HeT-A *subfamilies already integrated in its parental strain Oregon-R.

### Contribution of the different subfamilies to *HeT-A *transcription

In order to investigate the specific contribution of each *HeT-A *subfamily to the *HeT-A *transcriptome in both Oregon-R and GIII strains, we proceeded with the same analysis amplifying the *gag *and the 3'UTR fragments from RNA sources where *HeT-A *is actively transcribed (*see Methods*). RNA amplification was performed to distinguish sense and antisense strands, since antisense expression of *HeT-A *has previously been reported in germ line tissues [18]. Here, we find that antisense expression especially for the 3'UTR fragment is abundant and shows considerable size variability in larvae tissues (Figure 3). For the total number of analyzed RNA sequences for each strain see Table 1. We added the obtained sequences from both fragments to the existing DNA phylogenetic trees in order to be able to correlate expressed sequences with genomic copies (Figures 4 and 5) (blue Oregon-R and red GIII). Most expressed sequences nicely group with their genomic counterpart. This result is important because it demonstrates that our approach, in spite of having partial coverage, is good enough to study the *HeT-A *content and variability on these two strains. If our coverage had been too low to carry out these studies, we would expect to find a fair number of expressed or genomic sequences with no counterpart.

The first result from integrating the genomic and the transcriptomic data for both fragments (Figures 4 and 5) indicates that, with the exception of subfamily **I**, all existing subfamilies of both strains are expressed. Figures 4B and 5B allow a graphical view of the relative expression of the different subfamilies. Subfamilies **A **and **H **stand out as the ones with higher expression rate in both strains. Interestingly, subfamily **A **has a low copy number in both GIII and Oregon-R, suggesting the recent birth of this subfamily or its low transposition efficiency. Subfamilies **C **(with the exception of the 3'UTR fragment in GIII) and **G **would be an example of the opposite situation, the expression detected for these subfamilies is lower than expected for the genomic copies integrated in these genomes, nevertheless the differences in this case are subtle and could be explained by level of coverage of this approach.

A few *HeT-A *defective copies have been found outside the telomeres in the heterochromatic region of the III and Y centromeres [21-23]. Although the centromeric copies have not been previously classified in subfamilies, they all show 99% or higher sequence identity with the sequences from subfamilies **I **and **J **demonstrating that the 3'UTR primers used in this study are able to amplify sequences belonging to subfamilies **I **and **J**. Nevertheless, these copies are expressed at a very low level since we have only detected one sequence out of 44 for the **J **subfamily in Oregon-R and no sequence for the **I **subfamily. This result indicates that *HeT-A *expression coming from other genomic locations outside telomeres is very limited demonstrating that the expression of the *HeT-A *retrotransposon comes almost exclusively from the telomeres. Again, most subfamilies are expressed similarly in both stocks, supporting the evidence that the major *HeT-A *content in GIII comes from a proportional increase of the *HeT-A *subfamilies already present in the parental genetic background, Oregon-R.

Because we have also included in our analysis the *HeT-A *sequences from the two assembled telomeres (Additional file 1, and in black Figures 4A and 5A), and because in all cases these sequences group together with the newly identified *HeT-A *copies from our study, we can suggest that the *HeT-A *content in these two strains, Oregon-R and GIII does not differ substantially from the strain used in the genome sequencing project (isogenic strain 2057 yellow (*y^1^*); cinnabar (*cn^1^*) brown (*bw^1^*) speck (*sp^1 ^*)).

Finally, it is also important to mention that all the analyzed RNA sequences from the *gag *fragment are free of frame-shifts and other mutations resulting in *stop codons*, suggesting that all expressed sequences containing a *gag *fragment could produce a putative functional Gag protein. The phylogenetic relationships of the protein sequences corresponding to the DNA sequences for the *gag *fragment were analyzed and resulted very similar to the ones presented in Figure 4A (data not shown).

### Antisense *HeT-A *expression

While sense amplification is similar for both regions, antisense amplification for the 3'UTR fragment is highly abundant and heterogeneous (Figure 3C). On the contrary, antisense transcription for the *gag *fragment is nearly undetectable in Oregon-R and very poor in GIII (Figure 3C). Therefore, antisense transcription was only considered for the 3'UTR region and the number of sequences analyzed in this study is shown in Table 1.

**Figure 6:**
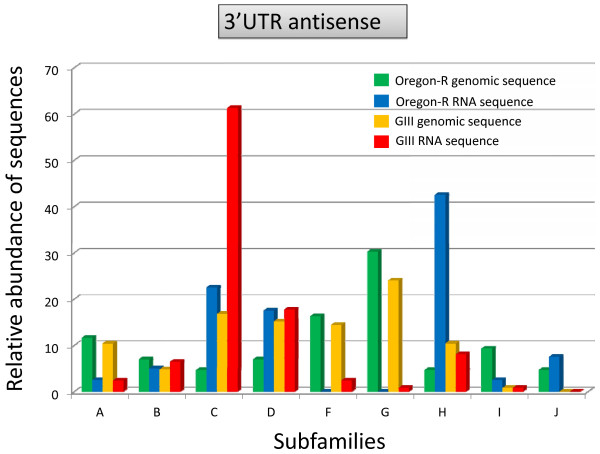
**Analysis of the antisense sequences amplified for the 3'UTR fragment**. All amplified sequences from genomic DNA of Oregon-R and GIII (bars in green and orange, respectively) and from total RNA from 3^rd ^instar larvae of Oregon-R and GIII (bars in blue and red, respectively) were used. The bar chart represents the relative abundance of each type of sequence for each subfamily. While the genomic sequences are the same than in the previous figure and the subfamily number is maintained, the amplified antisense RNA sequences show a different proportion for each subfamily. Note the case of subfamily C in GIII, which represents the ~60% of all the antisense *HeT-A *expressed sequences in this strain.

Figure 6 reflects the relative amount of the antisense transcripts belonging to the different subfamilies. As shown in Figure 6, subfamily **C **in the case of GIII (60%) and subfamily **H **in the case of Oregon-R (40%) are the main producers of 3'UTR antisense transcripts. The comparison between the data from Figure 6 with Figure 5B, shows that these subfamilies are also actively transcribed in the sense strand in GIII and Oregon-R respectively. Other subfamilies show a lower but also similar transcriptional level from both strands like subfamilies **B **and **D**, and finally some subfamilies have almost no antisense expression, subfamilies **F **and **G**, that behave otherwise in the sense strand, or subfamilies **I **and **J **which have very low expression from both strands. As mentioned before, the sequences from subfamilies **I **and **J **belong to defective copies located outside the telomeres and this study demonstrates that their contribution to the total antisense pool of *HeT-A *transcripts, as it was also the case for sense transcription, is very low (1 out of 124 of subfamily **I **in GIII, and 1 out of 40 of subfamily **I **and 3 out of 40 of subfamily **J **in Oregon-R).

**Figure 7:**
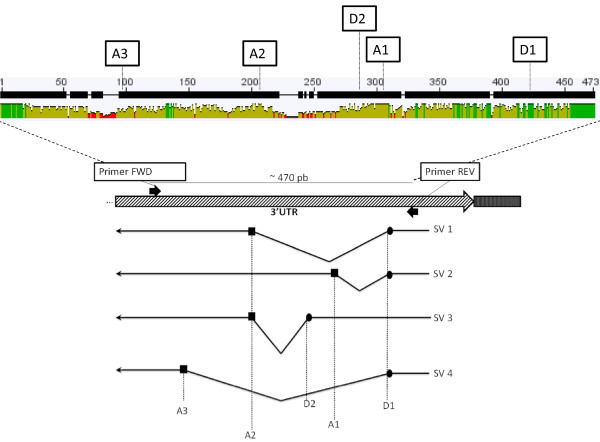
**Splice donor and acceptor analysis**. Top: consensus sequence obtained from the alignment of all the 3'UTR genomic sequences (total number: 168) indicating the identity level below each position (green: highest, red: lowest). The different donor (D) and acceptor (A) positions are indicated. Bottom: schematic representation of the different splicing variants. Dash arrow represents the *HeT-A *3'UTR. Black arrows indicate the primers used for amplification. Underneath, bullets identify splicing donors (D) and squares identify acceptors (A).

**Figure 8:**
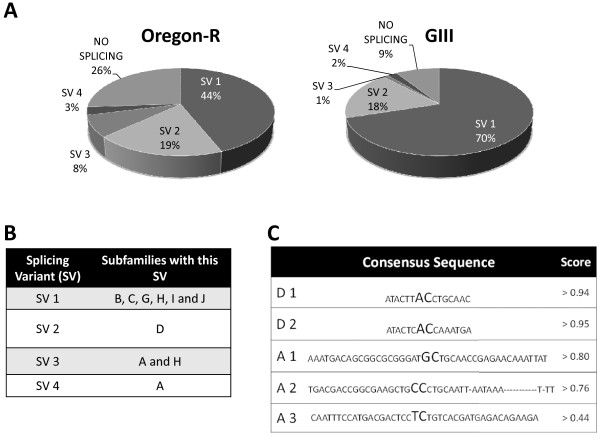
**Analysis of the different splicing variants from the antisense 3'UTR sequences**. **A**. Proportion of each splicing variant (SV) found in the analyzed strains. **B**. Table summarizing the splicing variant usage by each subfamily. **C**. Consensus sequence (reverse strand) for each donor (D) and acceptor (A). Splice site is indicated by two bigger letters. The NNSPLICE 0.9 score for each splice site is also indicated.

Antisense transcripts for the 3'UTR are highly variable in size (Figure 3C). After cloning and sequencing, we demonstrate that the size variability is due to several splicing processes to which most antisense transcripts (91% in GIII and 74% in Oregon-R) are subjected. Surprisingly, no sense transcript was found to be spliced. At least four different types of splicing in the 3'UTR fragment exist, being splicing variant 1 and 3 similar to those previously described [18] and splicing variants 2 and 4 here reported for the first time (Figure 7). Although splicing variant 1 is the most common splicing in both strains (70% in GIII and 44% in Oregon-R), the relative contribution of different subfamilies to each variant is different in both stocks (Figure 8A and 8B). In Oregon-R subfamily **H **is the most abundant, while in GIII subfamily **H **does not even represent the 10% of total antisense expression (Figure 6). All splice sites can be predicted by automated software with high score (Figure 8C) and they show higher conservation than the surrounding sequences (Figure 7). Importantly, splice donor 1 is present in all sequences, even if some are occasionally not processed. In GIII exists even a stronger bias of antisense transcription of the different subfamilies, being subfamily **C **accountable for more than 60% of the total antisense transcripts in this strain.

## Discussion

Intriguingly, the main component of Drosophila telomeres, the *HeT-A *retrotransposon, has the tendency to change faster than expected for a gene with an essential function and even for a retroelement [5]. Because telomere extension in Drosophila strongly depends on transpositionally capable *HeT-A *copies, it is important to better characterize the extent and the nature of this variability. We have attempted to classify this variability in different subfamilies and integrate our findings with previously reported results [6,7]. We show that the *HeT-A *sequence is even more variable than previously reported. As expected from this higher variability, we have identified at least nine different *HeT-A *subfamilies, five of which had not been reported previously. Our results also point out to the existence of some constraints to this rapid sequence change. Although the rate of sequence change is remarkable, we find in the GIII strain, with abnormally long telomeres and a ten fold difference in *HeT-A *copy number, the same variability reflected in a similar number and genetic distance among subfamilies. Although there might have not been enough time for the GIII and the Oregon-R stocks to show substantial variability, we believe that these similar patterns of distribution of the GIII and Oregon-R sequences into subfamilies could suggest that certain constraints are acting to keep the same scenario in both strains. Similarly, as shown in Figure 4A, the sequence for the *D.yakuba HeT-A gag *gene behaves as an outgroup verifying the existence of a threshold that keeps the identity of the sequences within *HeT-A *from *D.melanogaster*. Because of the function with which *HeT-A *is entitled, this retroelement must interact with different pathways and cell components at different levels. The requirements for such interactions may constitute the basis for some sequence change constraints. The resulting sequence dynamics could be explained by a putative genetic conflict in which the *HeT-A *retrotransposon would be locked together with Drosophila telomeres. In this scenario, although *HeT-A *would be constantly domesticated to fulfill the function of telomere replication, it would try to keep its parasitic nature by making the necessary changes to escape the telomere targeting and regulation imposed by other cellular components. The balance of these forces would result in the observed pattern of variability of the different *HeT-A *subfamilies. Alternatively, *HeT-A *might have only few functional domains that are under negative selection, and all changes in the rest of the sequence that do not impede its ability to transpose at telomeres will translate into the birth of a new subfamily.

Although the strain used to assemble the telomeres (2057 see below) [7,13] is different from the ones used in this study, and therefore to know the exact cytological position inside the telomeres it would be necessary to map all the sequences from this study at the telomeres of the Oregon-R and the GIII stocks, we have found high similarity among the sequences from the three strains suggesting a common trend at the level of transcription and genomic organization. Because cloning, sequencing and mapping the telomeres for any particular stock is highly problematic, and has not even been successfully achieved to completion for the sequenced stock (only two telomeres are assembled for the isogenic strain 2057 yellow (*y^1^*); cinnabar (*cn^1^*) brown (*bw^1^*) speck (*sp^1^*)), we were forced to compare our results with the only mapped telomere sequences (Additional file 1). Our studies have revealed the presence, at both genomic and transcriptomic level, of sequences that perfectly match *HeT-A *full length and truncated copies from the two unique assembled telomeres (Additional file 1 and sequences in black in Figures 4 and 5). The assembled data shows that these particular copies of *HeT-A *are located both in different telomeres and inside a telomere, at both distal and proximal regions. Understanding if the *HeT-A *copies capable of transposition were located in a specific region of the telomere was until now a puzzling question. Taking into account that all subfamilies are transcribed, but not all of them are likely to be present at the same telomeric position or in full-length copies, we suggest that *HeT-A *elements are expressed regardless of their position and whether they are complete or not. However, it is possible that differences in RNA levels between subfamilies could still depend on how many elements of a subfamily are terminal *vs*. proximal and full-length *vs*. truncated. The putative simultaneous presence of mRNAs from the full length copies of *HeT-A *(transposition intermediates) and mRNAs from truncated copies could be explained by a complex regulatory mechanism in which full length and small RNAs (sense and antisense) would interact and control *HeT-A *transposition and the telomeric chromatin (see below). Work by Shpiz et al. [18] demonstrated a direct relationship between the presence of antisense transcripts of *HeT-A *in ovaries and the rasiRNA pathway. Because the antisense *HeT-A *transcripts reported here do not come from germ line tissues, we suggest that the antisense transcription of *HeT-A *in somatic tissues could also be involved in a mechanism of RNA interference working in somatic cells. It is worth mentioning that we have detected expression of sequences with high identity with truncated *HeT-A *copies located in pericentromeric heterochromatin [21-23], but the low level of expression of these copies in comparison with the ones putatively located at the telomeres rules out a major contribution of the pericentromeric copies in the regulation of *HeT-A *expression. Thereby, we propose that the truncated copies of *HeT-A *retrotransposon at the telomeres could act in a similar way than truncated copies from *master loci *such as *flamenco*, acting to control transposition of other Drosophila retrotransposons (*gypsy, Zam *and *Ideafix*) [24,25]. Nevertheless, an important difference exists between *HeT-A *and the majority of Drosophila retrotransposons, the number of full length copies of *HeT-A *per genome is much higher. The maintenance of a good reservoir of full length copies is probably related to its involvement in building the telomeres, and might require a slightly different regulation than other retrotransposons with no apparent function in the genome, which allows the integration of *HeT-A *copies whenever telomeres are extended.

An alternative and not exclusive explanation for our observations, is that the different small RNAs of the *HeT-A *copies have additional functions besides the regulation of its own transcription. Recently the world of the non-coding RNAs is being expanded with new functions often involved in chromatin structure and regulation. Examples like the *Xist *RNA involved in mammalian dosage compensation or, of course, the TERRA RNA in telomere regulation, and the capacity of other non-coding RNAs to recruit histone modifying enzymes like the *HOTAIR *and the Ezh2 enzyme for regulation of the human HOXC [16,26,27], allow us to suggest that some of the *HeT-A *RNAs could have important additional roles at Drosophila telomeres. In this direction, a recent paper has demonstrated how some *HeT-A *small RNAs coming from the telomeres are essential to recruit the capping complex that protects Drosophila telomeres [19]. In order to transpose properly, a *HeT-A *copy has to be entirely transcribed from the sense strand. Transcription begins at the 3'UTR end of the immediately upstream *HeT-A *element and extents along the complete downstream copy [28]. On top of the sense transcription, *HeT-A *is also transcribed in the antisense strand in ovaries [18] and in larvae tissues (this study). Interestingly, we have found a large number of antisense transcripts of *HeT-A*, most of which are spliced through a small number of very conserved splicing alternatives. The antisense transcripts mainly come from the 3'UTR since only a few transcripts seem to extent into the *gag *gene (Figure 3C). Although the fewer *gag *antisense transcripts could be explained by the major number of 3'UTR regions integrated in the genome than copies with the *gag *gene, other factors like the presence of terminating signals may explain better the different level of antisense expression. The fact that, in spite of the high variability the antisense transcripts strongly conserve the splice donors and acceptors sites suggests that this processing is important and points once more to a putative functional role. Importantly, the major difference that we have found between a strain with abnormally long telomeres, GIII, and a wild type background, Oregon-R, concerns the antisense transcription. In GIII, the antisense transcript coming from the 4R6268 *HeT-A *copy (or subfamily C relatives) represents more than 60% of the total pool of antisense transcripts in this stock (Figure 6). This *HeT-A *copy has not massively integrated in the past in any of the two strains and is poorly transcribed from the sense strand. Therefore, it is tempting to suggest that this transcript could have an effect on *HeT-A *regulation and we could even speculate that a possible distortion in *HeT-A *regulation caused by the higher presence of this particular spliced antisense transcript could in part explain the longer telomere phenotype of the mutant background in the GIII stock. Further studies should address the reasons of the higher antisense expression of this particular copy and the possible consequences for *HeT-A *regulation and telomere biology in Drosophila.

## Conclusions

The study presented here demonstrates that the sequence variability of the *HeT-A *retrotransposon is much higher than previously reported defining at least nine different *HeT-A *subfamilies. Most of these subfamilies are actively being transcribed and likely contribute to the telomere extension in different *Drosophila melanogaster *strains. *HeT-A *is one of the few non-LTR retrotransposons capable of maintaining different subfamilies simultaneously transposing actively. Finally, the presence of a highly conserved splicing process only in the antisense transcripts of the 3'UTR region points to a putative regulatory, functional or structural role for the *HeT-A *RNA.

## Methods

### Drosophila stocks

All Drosophila stocks were maintained at 25°C on a standard yeasted corn meal-molasses medium. Oregon-R is a standard laboratory wild type strain. The Gaiano III (GIII) stock carries the third chromosome from the Gaiano strain (with the *Tel-1 *mutation) in an Oregon-R background [20].

### Genomic DNA and total RNA extraction

5 female and 5 male adult flies were collected from each strain and used to perform the genomic DNA extraction. The frozen flies were pounded with a pellet pestle in lyses buffer containing 0.1 M Tris-HCl, 0.1 M EDTA and 1% SDS; and left to incubation during 30 minutes. The resulting supernatant after centrifugation was precipitated with 1 M potassium acetate and 0.5 volumes of isopropanol and finally washed with ethanol and resuspended with milliQ H_2_O. The DNA concentration and quality were checked using NanoDrop^® ^ND-1000.

5 female and 5 male 3^rd ^instar larvae were collected from each strain and used to perform the total RNA extraction (RNeasy^® ^Mini Kit, Qiagen ref.74104). DNAse I treatment as follows: once with RNase-Free DNase set (Qiagen ref.79254) on-column, as manufacturer instructions, and twice for 3 hours with the same DNAse I in solution, as manufacturer instructions. The RNA concentration and quality were checked using NanoDrop^® ^ND-1000.

### Quantitative PCR (qPCR)

The primers used to quantify the genomic copy number in both strains were: HeT-A Real Time F (5' ACAGATGCCAAGGCTTCAGG 3') and HeT-A Real Time R (5' GCCAGCGCATTTCATGC 3') that bind to the 3'UTR of *HeT-A*. In order to normalize the data, we also quantified the genomic copy number of *actin *in all samples using the primers: Actin F (5' GCGCCCTTACTCTTTCACCA 3') and Actin R (5' ATGTCACGGACGATTTCACG 3'). The qPCR was carried out using the iQ SYBR Green super mix (Biorad) in iQ Real-Time PCR thermal cycler (Biorad), following standard qPCR protocols. The data was analyzed with the iQ software (Biorad).

### *HeT-A *genomic and RNA amplification, cloning and sequencing

The primers used to amplify the *gag *fragment were: GAG F (5' GGCGCCAAAAGCAGCATC 3') and GAG R (5' AACGTCCGGCTTGGGGTT 3'). The primers used to amplify the 3'UTR fragment were: 3UTR F (5' GCTCCAAGCTGCCAATCC 3') and 3UTR R (5' GCCAGAAGGACGGAAGCAC 3'). Genomic amplification was performed by standard PCR using Biotools DNA Polymerase (Cat Nº 10047). RNA amplification was performed by RT-PCR (Transcriptor One-Step RT-PCR Kit (Roche ref. 04655877001)) as directed by the manufacturer, specific for sense or antisense transcript amplification. Amplified products were directly ligated into pSTBlue-1 plasmid, using the AccepTor™ Vector Kit (Novagene ref. 70595-3). Plasmid DNA was purified by standard alkaline miniprep protocol. Insert presence was checked by EcoRI (Fermentas, ref. #ER0271) restriction. The plasmid DNA was sequenced by the Value Service of Macrogen Inc. (Korea) using the T7 promoter primer.

### Taq Polymerase error test

The error test of the Biotools DNA Polymerase (Cat Nº 10047) was performed amplifying, cloning and sequencing an already known clone. Eleven sequences comprising 459 nucleotides were analyzed and no error was found.

### Sequence analysis

The sequence alignments were carried out using MAFFT 6.833b program [29]. The phylogenetic trees were constructed using RAxML 7.2.8 program [30] performing 1000 bootstrap analysis for each tree. Clustering of sequences into subfamilies was done so that any sequence within a subfamily had no more than 10% of nucleotide changes with respect to another, excluding gaps. The search for splice sites in the obtained 3'UTR antisense transcripts was performed using the NNSPLICE 0.9 software [31]. The identity plot for the sequence alignment in Figure 7 was obtained using Geneious 4.8.3 software [32].

## List of abbreviations

MY: Millions of Years; ORF: Open Reading Frame; TERRA: Telomeric-Repeat containing RNA; qPCR: quantitative Polymerase Chain Reaction; UTR: Un-Translated Region; EDTA: Ethylenediaminetetraacetic acid; SDS: Sodium Dodecyl Sulfate; bp: base pairs.

## Competing interests

The authors declare that they have no competing interests.

## Authors' contributions

DP performed research and wrote the paper, EL-P and ML-P performed research and EC conceived, directed the study and wrote the paper. All authors read and approved the final manuscript.

## Supplementary Material

Additional file 1 **Schematic representation of the assembled data from Drosophila telomeres (source: **http://flybase.org**)**. XL, left telomere chromosome X (aprox. 20 Kb). 4R, right telomere chromosome 4 (aprox. 70 Kb). Names of *HeT-A *copies are indicated. Domains of complete *HeT-A *element, 5'UTR, *gag *gene and 3'UTR (dotted, smooth and lined boxes, respectively) are indicated. Click here for file

Additional file 2 ***HeT-A *sequences previously available on databases**. Names and accession numbers are given. X indicates if *HeT-A *elements are complete and if the sequence has been used in the *gag *and/or 3'UTR fragment analysis. Click here for file
