# Supplementary material for: Transcriptional analysis of the HeT-A retrotransposon in mutant and wild type stocks reveals high sequence variability at Drosophila telomeres and other unusual features
Source: BMC Genomics. 2011 Nov 23;12:573. doi: 10.1186/1471-2164-12-573 (PMC3235214; doi:10.1186/1471-2164-12-573)
Supplement: Additional file 1 — Schematic representation of the assembled data from Drosophila telomeres (source: http://flybase.org). XL, left telomere chromosome X (aprox. 20 Kb). 4R, right telomere chromosome 4 (aprox. 70 Kb). Names of HeT-A copies are indicated. Domains of complete HeT-A element, 5'UTR, gag gene and 3'UTR (dotted, smooth and lined boxes, respectively) are indicated. [file 1471-2164-12-573-S1.PPT]

## Slide 1
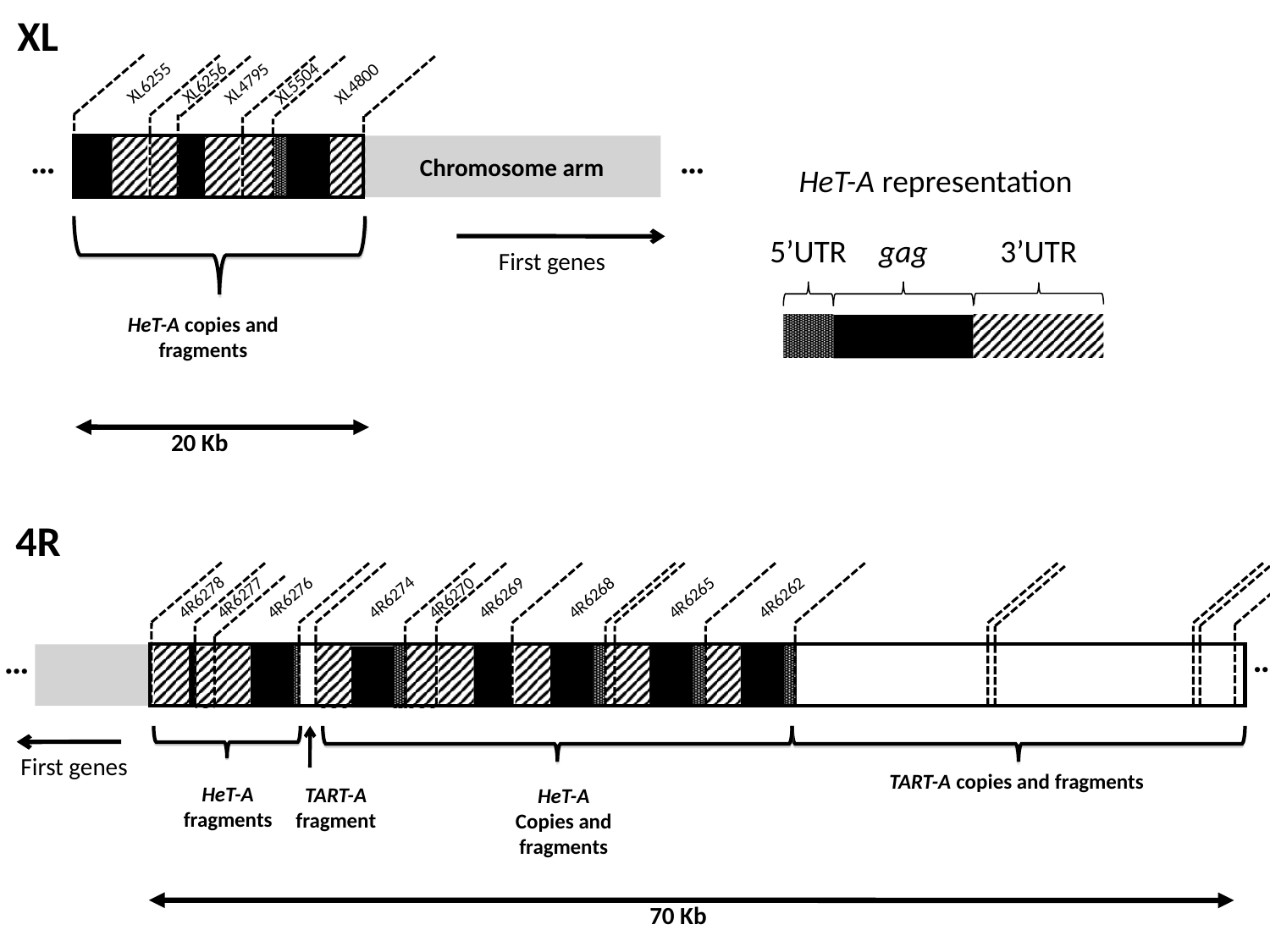

XL
XL6255
XL6256
XL5504
XL4795
XL4800
…
…
Chromosome arm
HeT-A representation
5’UTR
gag
3’UTR
First genes
HeT-A copies and fragments
20 Kb
4R
4R6278
4R6274
4R6262
4R6277
4R6276
4R6270
4R6269
4R6268
4R6265
…
…
First genes
HeT-A
fragments
HeT-A
Copies and fragments
TART-A copies and fragments
TART-A
fragment
70 Kb
