# Supplementary material for: Transcriptional analysis of the HeT-A retrotransposon in mutant and wild type stocks reveals high sequence variability at Drosophila telomeres and other unusual features
Source: BMC Genomics. 2011 Nov 23;12:573. doi: 10.1186/1471-2164-12-573 (PMC3235214; doi:10.1186/1471-2164-12-573)
Supplement: Additional file 2 — HeT-A sequences previously available on databases. Names and accession numbers are given. X indicates if HeT-A elements are complete and if the sequence has been used in the gag and/or 3'UTR fragment analysis. [file 1471-2164-12-573-S2.PPT]

## Slide 1
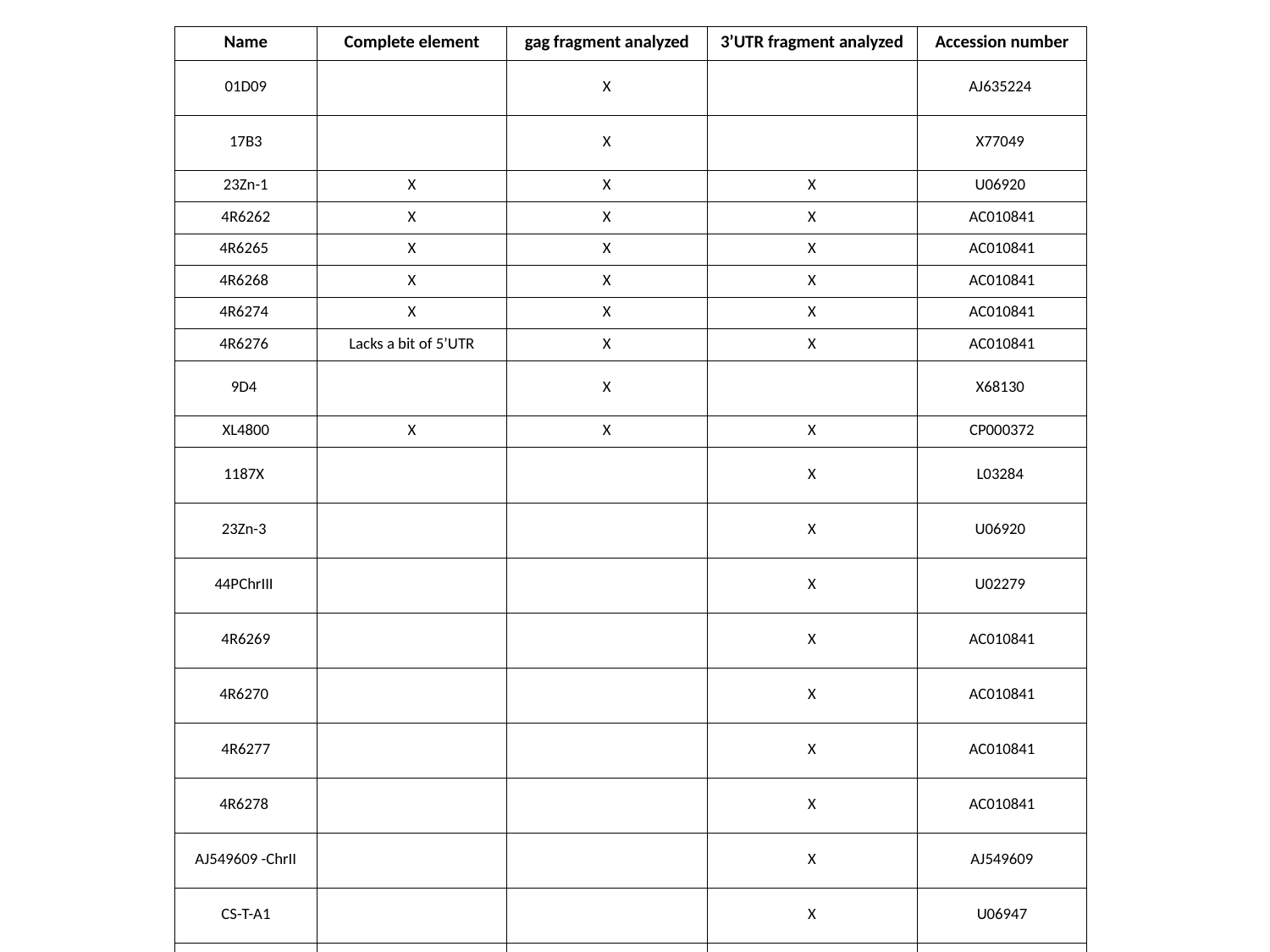

| Name | Complete element | gag fragment analyzed | 3’UTR fragment analyzed | Accession number |
| --- | --- | --- | --- | --- |
| 01D09 | | X | | AJ635224 |
| 17B3 | | X | | X77049 |
| 23Zn-1 | X | X | X | U06920 |
| 4R6262 | X | X | X | AC010841 |
| 4R6265 | X | X | X | AC010841 |
| 4R6268 | X | X | X | AC010841 |
| 4R6274 | X | X | X | AC010841 |
| 4R6276 | Lacks a bit of 5’UTR | X | X | AC010841 |
| 9D4 | | X | | X68130 |
| XL4800 | X | X | X | CP000372 |
| 1187X | | | X | L03284 |
| 23Zn-3 | | | X | U06920 |
| 44PChrIII | | | X | U02279 |
| 4R6269 | | | X | AC010841 |
| 4R6270 | | | X | AC010841 |
| 4R6277 | | | X | AC010841 |
| 4R6278 | | | X | AC010841 |
| AJ549609 -ChrII | | | X | AJ549609 |
| CS-T-A1 | | | X | U06947 |
| CS-T-A2 | | | X | U06947 |
| HTChrIII | | | X | AJ243350 |
| RT394 | | | X | M84201 |
| RT473 | | | X | M84200 |
| XL4795 | | | X | CP000372 |
| XL5504 | | | X | CP000372 |
| XL6255 | | | X | CP000372 |
| XL6256 | | | X | CP000372 |
